# Clinical value of the Systemic Inflammatory Response Index for predicting acute pancreatitis severity in Vietnamese setting

**DOI:** 10.1002/jgh3.13101

**Published:** 2024-06-14

**Authors:** Yen H T Dao, Tien M Huynh, Duy T Tran, Phat T Ho, Thong D Vo

**Affiliations:** ^1^ University of Medicine and Pharmacy at Ho Chi Minh City Ho Chi Minh City Vietnam; ^2^ University Medical Center Ho Chi Minh City Ho Chi Minh City Vietnam; ^3^ 30‐4 Hospital Ho Chi Minh City Vietnam; ^4^ Cho Ray Hospital Ho Chi Minh City Vietnam

**Keywords:** acute pancreatitis, Bedside Index for Severity in Acute Pancreatitis, prognosis, Systemic Inflammatory Response Index, Vietnam

## Abstract

**Background and Aim:**

Accurate prediction of severe acute pancreatitis (SAP) is crucial for timely intervention. This study focuses on the Systemic Inflammatory Response Index (SIRI) to assess its clinical value in predicting the severity of AP in the Vietnamese context.

**Methods:**

A cross‐sectional prospective study was conducted with acute pancreatitis patients at a national hospital in Ho Chi Minh City. The patients were classified into nonsevere and severe groups, and the clinical characteristics were analyzed. The predictive abilities of SIRI, calculated using neutrophil × monocyte/lymphocyte, was assessed for predictive abilities. Multivariate regression and receiver operating characteristics (ROC) curves evaluated the prognostic factors and predictive accuracy.

**Results:**

Among 207 patients, 78.7% had nonsevere AP, and 21.3% had SAP. The severe group exhibited a significantly higher median SIRI (12.0) than the nonsevere group (4.9) (*P* < 0.001). Multivariate regression identified SIRI (odds ratio [OR] = 1.623) as an independent predictor of SAP. The ROC curve determined a SIRI cutoff of 7.82 with an area under the curve (AUC) of 0.737. Combining the SIRI and Bedside Index for Severity in Acute Pancreatitis (BISAP) score improved the predictive ability (AUC = 0.820) with increased sensitivity (90.91%) (*P* < 0.001).

**Conclusion:**

SIRI, particularly when combined with the BISAP score, shows significant potential to predict SAP severity in the Vietnamese clinical setting, providing valuable information for effective patient management.

## Graphical abstract

This study evaluates the Systemic Inflammatory Response Index (SIRI) as a predictor of severe acute pancreatitis (SAP) in a Vietnamese clinical setting. Among 207 patients, a higher median SIRI was significantly associated with SAP. Multivariate regression confirmed SIRI as an independent predictor, and ROC analysis highlighted a cutoff of 7.82 (AUC 0.737). Combining SIRI with the BISAP score enhanced predictive accuracy (AUC 0.820) and sensitivity (90.91%), underscoring its clinical utility for timely intervention.

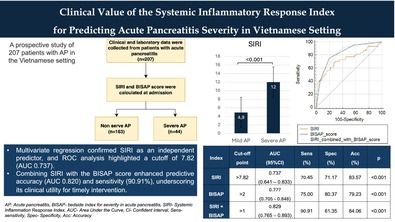

## Introduction

Acute pancreatitis (AP) represents an acute inflammatory disease that affects the pancreas, contributing to an increasing burden on global healthcare systems.^1^ The incidence of AP has shown an average annual increase of 3.07% between 1961 and 2016, underscoring the urgency of effective management strategies. This condition is clinically classified into mild, moderately severe, and severe categories, with the mortality rate of mild cases being less than 1%.^2^ However, 15–25% of patients progress to moderately severe or severe AP, increasing the complications and mortality risks. The timely initiation of treatment significantly influences patient outcomes, necessitating accurate diagnosis and early prediction of disease severity.^3^ Various scoring systems have been developed to assess the severity and prognosis of AP, such as Ranson criteria, acute physiologic assessment and chronic health evaluation II (APACHE II) score, Bedside Index for Severity in Acute Pancreatitis (BISAP) score, systemic inflammatory response syndrome (SIRS), hematocrit (Hct), and C‐reactive protein (CRP). Each tool has distinct advantages and disadvantages, which contribute to the complexity of choosing the optimal predictive method. Despite their utility, none of these tools has been universally effective in predicting the severity of AP.^4^ A novel marker, Systemic Inflammation Response Index (SIRI), has emerged as a promising indicator reflecting the delicate balance between inflammation and the immune response of the body. Calculated from hematologic parameters, including neutrophil, lymphocyte, and monocyte counts, SIRI was first introduced in 2016 to predict survival in patients with pancreatic cancer patients post‐chemotherapy.^5^ Subsequently, in 2017, researchers adapted SIRI to predict the overall survival rates in patients with hepatocellular carcinoma. Since 2020, SIRI has been scrutinized for its role in the prediction of severe acute pancreatitis (SAP).^6^ In particular, SIRI is easy to calculate, cost‐effective, and relies solely on a complete blood count, eliminating subjectivity. Despite its potential, there are no available data on SIRI in Vietnam. This study aims to investigate the role of SIRI and its combination with the BISAP score at admission in predicting the severity of AP in the Vietnamese population.

## Methods

### Patient selection

Our prospective cross‐sectional study was conducted on patients diagnosed with AP admitted to the Department of Gastroenterology of Cho Ray Hospital in Ho Chi Minh City, Vietnam, from May 2023 to September 2023.

The inclusion criteria were patients 18 years or older, who met the diagnostic criteria for AP according to the Atlanta 2012 revised classification and who agreed to participate in the study. Exclusion criteria comprised patients with malignant tumors and hematologic, autoimmune, inflammation‐related, or chronic liver/kidney diseases, pregnancy, using corticosteroids or immunosuppressive drugs, insufficient data, chronic pancreatitis, and not agreeing to participate in the study.

### Data collection

We collected data on patients, including age and sex, etiology of AP, history and symptoms at admission, and laboratory data such as leukocyte, neutrophil, lymphocyte, monocyte count, Hct, amylase, lipase, triglyceride, CRP, blood urea nitrogen (BUN), and creatinine. Blood samples were obtained from all patients during hospitalization after fasting for at least 12 h. We also documented the complications of AP and the presence of pleural effusion.

Diagnosis of AP is based on the revised Atlanta classification of 2012, which requires two of the following three criteria: (i) abdominal pain consistent with acute pancreatitis (acute onset of persistent, severe, epigastric pain often radiating to the back); (ii) serum lipase activity (or amylase activity) at least three times greater than the upper limit of normal; and (iii) characteristic findings of acute pancreatitis on contrast‐enhanced computed tomography and less commonly magnetic resonance imaging or transabdominal ultrasonography.^7^

AP is classified into three levels: mild, moderately severe, and severe. Mild AP is characterized by the absence of organ failure and the absence of local or systemic complications. Moderately SAP is characterized by the presence of transient organ failure or local or systemic complications in the absence of persistent organ failure. SAP is characterized by persistent organ failure.^7^

SIRI is calculated based on hematological parameters at admission, including neutrophil, monocyte, and lymphocyte, using the formula SIRI = $\frac{\text{neutrophil}\times \text{monocyte}}{\text{lymphocyte}.}$.

Laboratory data are obtained using the machinery system at Cho Ray Hospital.

The BISAP score is composed of five different factors, each of which contributes one point to the overall score based on the fulfillment of their respective criteria. These factors include a BUN measurement that exceeds 25 mg/dL, a compromised mental state indicated by a Glasgow Coma Scale (GCS) score below 15 (impaired mental status), a SIRS score that equals or exceeds 2, an age of over 60, and the detection of pleural effusion on chest radiograph or CECT scans. The scores are obtained at admission and, if necessary, completed within 48 h. A high BISAP score means the score is equal to or greater than 3.^8^

### Sample size calculation

We used the formula $n_{D}=n_{\text{non}‐D}\geq \frac{Z_{1-\frac{\alpha }{2}}^{2}V_{\mathrm{AUC}}}{d^{2}}$, where $n_{D}$ is the number of patients with SAP and $n_{\text{non}‐D}$ is the number of patients without SAP. α=0.05, *d* = 0.1, and area under the curve (AUC) is 0.890 (according to a research by Biyik *et al*. about SIRI). The final sample size was 59 cases.

**Figure 1:**
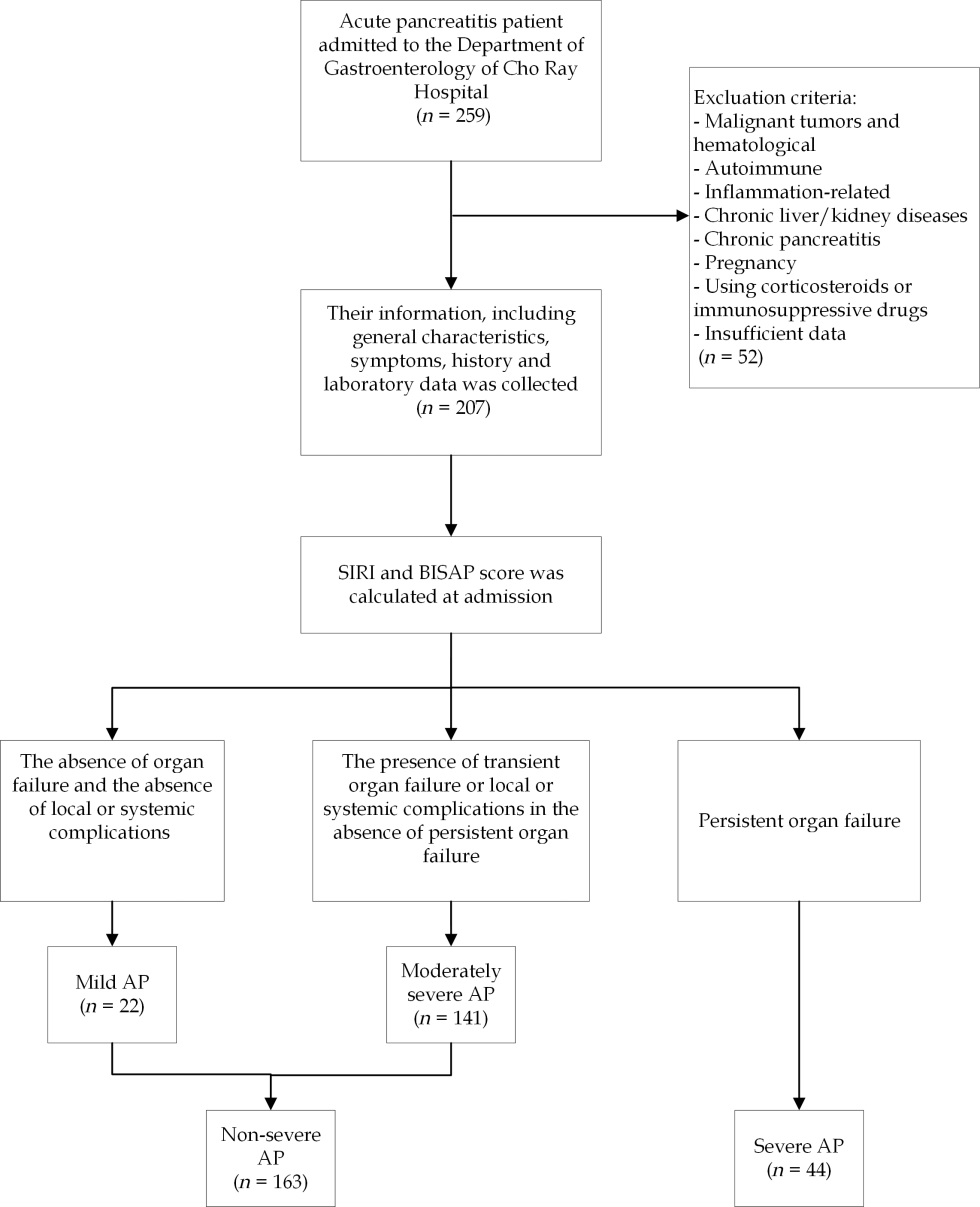
Flowchart of the study.

The flowchart of the study is illustrated in Figure 1.

### Ethics statement

The study received approval from the Ethics Committee for Biomedical Research at the University of Medicine and Pharmacy in Ho Chi Minh City (No. 506/HDĐD‐DHYD, signed May 4, 2023). Written informed consent was not required, in accordance with national legislation and institutional requirements. The study protocol adhered to the ethical principles of the Declaration of Helsinki.

### Statistical analysis

Data analysis was performed using Stata 14 software. Qualitative variables were expressed as frequencies and percentages, while quantitative variables were presented as mean ± SD for the normal distribution and median with interquartile range for the skewed distribution. Distribution normality was assessed with the Kolmogorov–Smirnov test. Comparison of qualitative variables employed the chi‐square or Fisher's exact test, and for quantitative variables, the *t* test or Mann–Whitney test was used based on distribution. Multivariate regression analysis explored the impact of factors on variables. ROC curves assessed the predictive ability of the SIRI and BISAP scores for SAP. Statistical significance was determined at *P* < 0.05.

## Results

In our study, 207 patients were admitted, consisting of 163 (78.7%) cases classified as nonsevere AP and 44 (21.3%) cases classified as SAP. The mean age of the study population was 45.6 ± 16.5 years, and 131 (63.3%) were male patients. Alcohol‐induced AP accounted for the highest proportion of cases at 29.0%. Detailed demographics, clinical characteristics, and laboratory data of patients are presented in Table 1 and Figure 1.

**Table 1:** Demographics, clinical characteristics, and laboratory data of the study population

Characteristic	Total (*n* = 207)	Nonsevere AP (*n* = 163)	SAP (*n* = 44)	*P*
Age (years)	45.6 ± 16.5	45.5 ± 16.9	45.8 ± 15.2	0.906
Male gender, *n* (%)	131 (63.3)	97 (59.5)	34 (77.3)	0.030
Etiology, *n* (%)				
Alcohol	60 (29.0)	47 (28.8)	13 (29.5)	0.926
Hypertriglyceridemia	59 (28.5)	45 (27.6)	14 (31.8)	0.583
Gallstones	56 (27.1)	48 (29.5)	8 (18.2)	0.135
Other	32 (15.4)	23 (14.1)	9 (20.5)	0.302
History, *n* (%)				
Acute pancreatitis	75 (36.2)	63 (38.7)	12 (27.3)	0.164
Diabetes	47 (22.7)	34 (20.9)	13 (29.6)	0.222
Using alcohol	121 (58.5)	91 (55.8)	30 (68.2)	0.140
Symptom, *n* (%)				
Pancreatic acute abdominal pain	198 (95.7)	158 (96.9)	40 (90.9)	0.098
Nausea and/or vomiting	139 (67.2)	107 (65.6)	32 (72.7)	0.375
Impaired mental status, *n* (%)	27 (13.0)	12 (7.4)	15 (34.1)	<0.001
Laboratory data				
WBC (G/L)	13.1 ± 5.0	12.4 ± 4.0	15.7 ± 7.0	0.003
Neutrophil (G/L)	10.8 ± 4.8	10.1 ± 4.0	13.4 ± 6.5	0.003
Monocyte (G/L)	0.7 ± 0.4	0.7 ± 0.3	0.9 ± 0.5	0.006
Lymphocyte (G/L)	1.3 ± 0.7	1.4 ± 0.7	1.2 ± 0.8	0.181
Hct (%)	39.3 ± 7.0	39.4 ± 6.5	38.9 ± 8.9	0.727
Amylase (U/L)	356.0 (149.5–623.8)	266.0 (109.0–592.0)	382.5 (151.2–686.0)	0.232
Lipase (IU/L)	616.4 (248.6–1309.6)	517.0 (171.8–1242.0)	671.6 (323.2–1411.2)	0.228
Triglycerides (mg/dL)	180.0 (112.5–664.5)	180.0 (109.0–569.0)	198.0 (113.0–664.5)	0.636
BUN (mg/dL)	12.0 (8.0–17.0)	11.0 (8.0–15.8)	16.5 (9.0–26.5)	0.001
Creatinine (mg/dL)	0.7 (0.6–0.9)	0.7 (0.6–0.9)	0.9 (0.7–2.1)	0.004
Complication, *n* (%)				
Acute peripancreatic fluid collection	179 (86.5)	140 (85.9)	39 (88.6)	0.636
Pancreatic necrosis	80 (38.7)	50 (30.7)	30 (68.2)	<0.001
Pancreatic pseudocyst	8 (3.9)	8 (4.9)	0 (0.0)	0.207
Walled‐off necrosis	7 (3.4)	5 (3.1)	2 (4.6)	0.642
Pleural effusion, *n* (%)	74 (35.8)	46 (28.2)	28 (63.6)	<0.001
Predicting tool				
High BISAP score, *n* (%)	65 (31.4)	32 (19.6)	33 (75.0)	<0.001
SIRI	5.5 (3.0–10.4)	4.9 (2.7–8.4)	12.0 (5.4–21.2)	<0.001

BISAP, Bedside Index For Severity in Acute Pancreatitis; BUN, blood urea nitrogen; Hct, hematocrit; SIRI, Systemic Inflammation Response Index; WBC, white blood count.

In Table 1, the SAP group showed a notable gender distribution imbalance, with a higher percentage of males (77.3%) compared with females (59.5%) (*P* = 0.030). Age, however, did not demonstrate a significant association with the severity of the disease (*P* = 0.906). The etiology of AP and the patient's history did not differ between the severe and nonsevere groups.

The SAP group showed a significantly higher prevalence of impaired mental status compared with the nonsevere AP group (34.1% *vs* 7.4%, *P* < 0.001). Analysis of complete blood count revealed a gradual increase in white blood cell (WBC) and neutrophil counts with the severity of the disease (*P* = 0.003 for both). On the contrary, monocyte and lymphocyte counts were not significantly correlated with disease severity (*P* = 0.006 and 0.181, respectively).

The serum levels of amylase and lipase were not correlated with increased severity of the disease (*P* = 0.232 and 0.228, respectively). On the contrary, BUN and creatinine levels in the SAP group were significantly higher than that in the nonsevere group (*P* = 0.001 and 0.004, respectively). Furthermore, the incidence of pancreatic necrosis was markedly higher in the SAP group compared with the nonsevere group (68.2% *vs* 30.7%, *P* < 0.001).

The BISAP score and SIRI were higher in the SAP group than in the nonsevere group. In the group of severe patients, 75.0% had a high BISAP score (3), while in the nonsevere group, it was only 19.6% (*P* < 0.001). The median SIRI was 12.0 (5.4–21.2) in the severe group, but was only 4.9 (2.7–8.4) in the other group (*P* < 0.001).

### Multivariate linear regression and logistic regression analysis for SIRI

Univariate regression analysis revealed statistically significant associations between SAP and various factors, including impaired mental status, creatinine levels, pancreatic necrosis, pleural effusion, SIRI, and high BISAP score (Table 2). However, in the subsequent multivariate regression analysis, only creatinine (OR = 1.090; 95% CI: 0.306–1.874; *P* = 0.006) and SIRI (OR = 1.623; 95% CI: 0.699–2.547; *P* = 0.001) retained significant associations with elevated rates of SAP (Table 3).

**Table 2:** Univariate regression analysis of factors predicting severe acute pancreatitis

Variable	OR	95% CI	*P*
Impaired mental status	1.873	1.016–2.730	<0.001
Creatinine	1.415	0.800–2.030	<0.001
Pancreatic necrosis	1.578	0.861–2.294	<0.001
Pleural effusion	1.493	0.791–2.196	<0.001
SIRI	1.666	0.946–2.385	<0.001
High BISAP score	2.508	1.724–3.292	<0.001

BISAP, Bedside Index for Severity in Acute Pancreatitis; CI, confidence interval; OR, odds ratio; SIRI, Systemic Inflammatory Response Index.

**Table 3:** Multivariate regression analysis of factors predicting severe acute pancreatitis

Variable	OR	95% CI	*P*
Impaired mental status	0.781	0.403–1.965	0.196
Creatinine	1.090	0.306–1.874	0.006
Pancreatic necrosis	0.876	0.006–1.758	0.052
Pleural effusion	0.891	0.113–1.896	0.082
SIRI	1.623	0.699–2.547	0.001
High BISAP score	1.007	0.139–2.154	0.085

BISAP, Bedside Index for Severity in Acute Pancreatitis; CI, confidence interval; OR, odds ratio; SIRI, Systemic Inflammatory Response Index.

**Figure 2:**
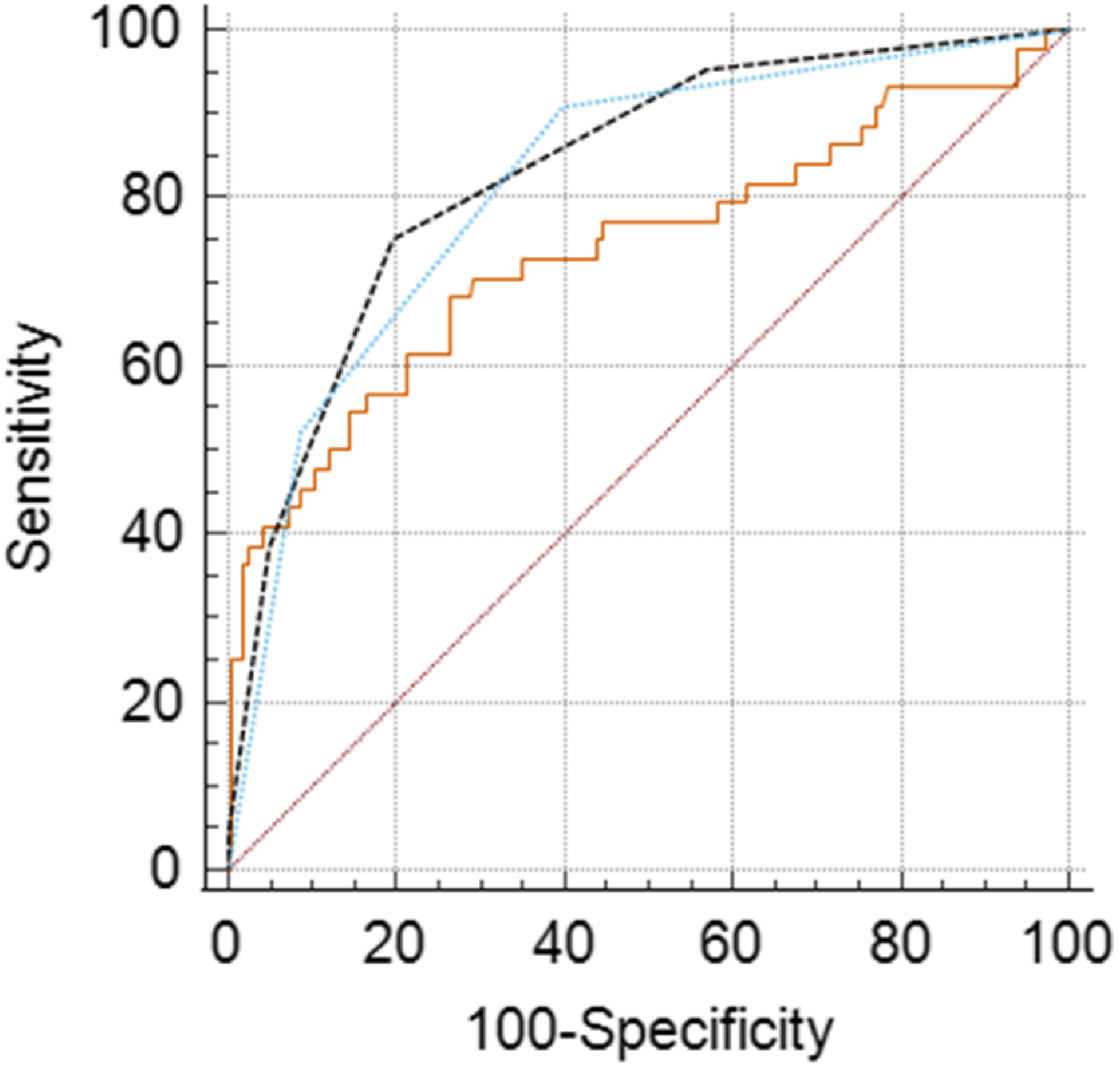
ROC curve of SIRI and BISAP score in predicting severe acute pancreatitis. BISAP, Bedside Index For Severity in Acute Pancreatitis; ROC, receiver operating characteristic; SIRI, Systemic Inflammatory Response Index. 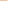, SIRI; 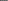, BISAP_score; 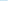, SIRI_combined_with_BISAP_score.

The receiver operating characteristics (ROC) curve analysis was conducted to assess the predictive capability of the SIRI, the BISAP score, and their combined application in forecasting SAP. The optimal cutoff point for SIRI was determined to be 7.82, while for the BISAP score, it was identified as 2 within our study population. When combining these two tools, the AUC was found to be 0.820 (95% CI: 0.756–0.885), surpassing the AUC values for each individual tool—0.737 (95% CI: 0.641–0.833) for SIRI and 0.777 (95% CI: 0.705–0.848) for the BISAP score. Furthermore, the combined use of SIRI and the BISAP score increased the sensitivity to 90.91%, as illustrated in Table 4 and Figure 2.

**Table 4:** ROC curves of tools in predicting severe acute pancreatitis

Index	Cutoff point	AUC (95%CI)	Sens (%)	Spec (%)	PPV (%)	NPV (%)	Accuracy (%)	*P*
SIRI	>7.82	0.737 (0.641–0.833)	70.45	71.17	73.08	65.52	83.57	<0.001
BISAP	≥ 3	0.777 (0.705–0.848)	75.00	80.37	29.80	70.31	79.23	<0.001
SIRI + BISAP	>1	0.829 (0.765–0.893)	90.91	61.35	20.72	79.41	84.06	<0.001

AUC, area under the curve; BISAP, Bedside Index for Severity in Acute Pancreatitis; CI, confident interval; NPV, negative predictive value; PPV, positive predictive value; ROC, receiver operating characteristics; Sens, sensitivity; SIRI, Systemic Inflammatory Response Index; Spec, specificity.

## Discussion

Our study marks the first pioneering investigation of this severity index for AP in Vietnam. We demonstrate that SIRI has significant potential to predict SAP. Furthermore, our findings suggest that the synergistic use of SIRI and BISAP can enhance the prognostic accuracy of AP in the Vietnamese context.

AP is a high‐stakes disease marked by sudden onset, rapid progression, and notable rates of mortality and morbidity. The evaluation of the prognosis is challenging, and current methods need more sensitivity and specificity. The recent focus on inflammatory markers for the prognosis of AP has highlighted SIRI reflecting immune status. SIRI, identified as a reliable systemic inflammatory biomarker, provides information on inflammation status. The crucial role of immune cells, particularly neutrophils, in the inflammatory response is integral to the pathogenesis of AP. Neutrophil recruitment triggers trypsinogen activation, escalating pancreatic damage. SIRI emerges as a promising tool for the prognosis of AP, shedding light on immune status and inflammatory processes.^9^ Monocytes, in conjunction with neutrophils, are pivotal innate immune cells in the pathogenesis of AP. Migrating to tissues, monocytes transform into macrophages, actively contributing to the early stages of AP. Infiltrating the pancreas, these macrophages become key producers of inflammatory mediators, including TNF‐α, IL‐1β, IL‐6, IL‐18, and MCP‐1, fostering a feedback loop that amplifies local inflammation. This cascade intensifies tissue damage, increasing the severity of AP. The involvement and differentiation of monocytes into macrophages highlight the intricate and dynamic role of innate immune cells in AP inflammatory processes.^10^ In addition to the crucial role of innate immunity, adaptive immunity is significant in the development of AP. AP is characterized by a marked decrease in T and B lymphocyte counts in peripheral blood. Early‐stage reductions in T and B lymphocytes are comparable, but in the late phase, B lymphocytes exhibit a more persistent decline than T lymphocytes. This sustained decrease in B lymphocyte count extends until the 30th day, underscoring the lasting impact of adaptive immune system changes in later AP stages.^11^, ^12^ Due to the complex nature of the pathogenesis of AP, it is impractical to predict the severity of the disease based on only one parameter. In particular, in SAP, there are significantly elevated neutrophil and monocyte counts alongside a marked decrease in lymphocyte count.^11^ The combination of hematological parameters related to inflammation, such as SIRI (neutrophil × monocyte/lymphocyte), is positive. Although there were limited studies on the role of SIRI in AP, recent research by Pedro Silva‐Vaz *et al*. (2021) revealed notable findings. The study demonstrated that SIRI at admission in the severe AP group measured 11.4 ± 4.9, significantly higher than the comparison group (*P* = 0.022). The AUC for SIRI to predict the severity of AP was 0.82, which emphasizes its potential as a prognostic indicator for the severity of AP.^13^ In 2022, a study by M. Biyik *et al*.^14^ involving 332 patients, SIRI at admission was significantly higher in SAP patients (*P* < 0.001). At a cutoff point of 3.72, SIRI demonstrated predictive precision for SAP, with a sensitivity of 92.0% and specificity of 59.3%, yielding an AUC of 0.782.^14^ Furthermore, a study by Yildiz *et al*.^15^ on 201 patients also found higher levels of SIRI in SAP patients (mean SIRI 11.19 ± 6.27 in SAP *vs* 3.12 ± 3.01 in nonsevere AP; *P* < 0.001). At a cutoff point of 4.83, SIRI exhibited strong predictive capability for SAP, with an AUC of 0.890, and sensitivity and specificity values of 83.8% and 80.0%, respectively.

In our research in Vietnam, we found a statistically significant elevation of SIRI at admission in patients with severe disease. Multivariate regression analysis revealed SIRI as an independent prognostic factor for SAP. At a cutoff point of 7.82, SIRI demonstrated a sensitivity of 70.45% and a specificity of 71.17%, with an AUC of 0.737 (95% CI: 0.641–0.833). Furthermore, combining SIRI with the BISAP score significantly increased the ability to predict disease severity to a good level (AUC = 0.820), achieving a sensitivity of 90.91% at a cutoff >7.82. This comprehensive approach offers promising prospects for improved prognostication in SAP. Table 5 provides a comparison between our study and others. Compared with others, the observed discrepancy in our study could be attributed to factors such as delayed patient presentation, variations in referral patterns from other institutions, differences in race and sex ratio, and variances in local primary treatment protocols. There are many factors that can affect the NLR and monocyte beyond AP. For example, men often have a higher NLR and monocyte count than females.^16^, ^17^ In our study, the percentage of men was 63.3%, surpassing the figures of Silva‐Vaz, Biyik, and Yildiz, where the male percentages were 31.0%, 45.5% and 52.8%, respectively. Furthermore, diabetic patients typically exhibit a higher NLR than the general population. In our study, we observed a higher prevalence of diabetic patients than Biyik, resulting in an elevated SIRI in our findings.^16^

**Table 5:** Study characteristics of the utilization of the Systemic Inflammatory Response Index (SIRI)

Author (year)	Country	Hospital	Type of study	*N*	Criteria of severity AP	Severe AP *n* (%)	Cutoff	Sens	Spec	PPV	NPV	AUC
Silva‐ Vaz (2021)^6^	Portugal	Department of General Surgery of the Hospital Amato Lusitano of Unidade Local de Saúde de Castelo Branco, University Teaching Hospital	Retrospective	117	Revised Atlanta classification	44 (21.3)	7.14	82	87	53	96	0.906
Biyik (2022)^14^	Turkey	Gastroenterology Clinic and Intensive Care Units of Necmettin Erbakan University, Meram Faculty of Medicine Hospital	Retrospective	332	Revised Atlanta classification	24 (7.2)	3.72	92	59.3	69.3	88.1	0.809
Yildiz (2023)^15^	Turkey	Health Science University Antalya Training and Research Hospital	Retrospective	201	Revised Atlanta classification	36 (17.9)	4.83	83.0	80	NR	NR	0.890
Our study (2023)	Vietnam	Department of Gastroenterology, Cho Ray Hospital	Prospective	207	Revised Atlanta classification	44 (21.3)	7.82	70.5	71.2	73.1	65.5	0.737

AP, acute pancreatitis; AUC, area under the curve; NPV, negative predictive value; NR, not reported; PPV, positive predictive value; Sens, sensitivity; Spec, specificity.

The study has several strengths that make it significant. It is the first prospective exploration of the correlation between SIRI and the diagnosis of AP in the Vietnamese population. Prospective studies generally offer a reduced potential for bias and confounding compared with retrospective studies, making our findings more reliable. The study suggests that SIRI may be a valuable index for assessing the severity of AP in Vietnamese patients, especially in emergency departments and primary healthcare facilities where resource constraints are common. It could potentially replace other challenging scoring systems in stratified patient care.

However, certain limitations should be considered. Our study was carried out in a single center, specifically a tertiary facility with a higher prevalence of severe AP cases, potentially limiting the generalizability of our findings to the broader population. Additionally, our focus on disease severity meant that we did not investigate other important clinical outcomes such as severe AP, length of hospital stay, or mortality rates. This lack of comprehensive exploration may affect the applicability of our findings to broader clinical contexts. In addition, our study only reported SIRI at admission, failing to capture its dynamic nature over time and its reflection of the inflammatory process. This limitation prevents us from determining the optimal detection time window for SIRI. Future multicenter studies with larger sample sizes could benefit from a dynamic evaluation of SIRI through serial follow‐ups, providing a more nuanced understanding of its role in predicting various outcomes.

In conclusion, SIRI at admission is closely correlated with SAP. As a readily accessible, rapid, and straightforward index, SIRI is a valuable tool for early patient stratification, enabling a proactive approach to monitoring and treatment. Combining SIRI with the BISAP score also achieves high performance in SAP predictive, paving the way for promising avenues in future research.

## Consent statement

Patient consent was obtained.

## Data Availability

The data and code supporting the findings of this study are available from the corresponding author upon reasonable request.
